# Psychophysiological Adaptations to Yoga Practice in Overweight and Obese Individuals: A Topical Review

**DOI:** 10.3390/diseases10040107

**Published:** 2022-11-17

**Authors:** Alexios Batrakoulis

**Affiliations:** Department of Physical Education and Sport Science, University of Thessaly, 42100 Trikala, Greece; abatrakoulis@uth.gr; Tel.: +30-24310-47018

**Keywords:** yoga, exercise, overweight, obesity, physiological responses, psychological responses

## Abstract

Physical activity has been documented as a foundational approach for weight management and obesity, improving several cardiometabolic and mental health indices. However, it is not clear whether yoga practice can induce beneficial improvements in anthropometric and body composition parameters, performance, metabolic health, and well-being among overweight/obese people. The aim of this topical review was to catalog training studies examining the psychophysiological responses to yoga interventions in order to detect which outcomes have been investigated, the research methods applied, and the conclusions. The inclusion/exclusion criteria were met by 22 published articles involving 1178 (56% female) overweight/obese participants. This brief review on yoga-induced adaptations demonstrates that this widely used meditative movement activity can meaningfully improve the vast majority of the selected markers. These beneficial alterations are focused mostly on various anthropometric and body composition variables, cardiovascular disease risk factors, physical fitness parameters, quality of life, and stress in previously inactive overweight/obese individuals. Instead, yoga-based physical exercise interventions investigating anxiety, depression, mood state, exercise enjoyment, affect valence, and adherence were limited. Further research should focus on the yoga intervention configuration and potential mechanisms behind favorable changes in various psychophysiological indices through large-scale, rigorously designed randomized controlled trials implementing long-term interventions in overweight/obese individuals.

## 1. Introduction

### 1.1. Obesity: An Urgent Global Epidemic

Obesity is a multifactorial chronic metabolic disease characterized by excess body fat that adversely predisposes to poor physiological and psychological health while developing various comorbidities [1]. The recently increasing prevalence of overweight and obese individuals at the global level has been reported as a major public health issue. In fact, more than one in two adults worldwide do not have a healthy weight, and the problem is currently bigger in the Western world where almost two in three are overweight or obese [2]. The economic burden of obesity will rise to nearly USD 2.0 trillion annually starting from 2025, inducing a serious economic and social impact worldwide [3]. Obesity is a complex health problem affected by genetic, lifestyle, environmental, physiological, and psychological factors with major consequences for society. Such a disease results in various lifestyle-related chronic diseases through several cardiometabolic complications as well as mental health abnormalities [4]. Interestingly, overweight and obese individuals are unlikely to show sufficient physical activity levels [5], while they demonstrate poor mental health [6] and low mobility as well as functionality [7]. Due to both low cardiorespiratory and musculoskeletal fitness levels, individuals with unhealthy weight struggle to participate in physical exercise on a regular basis [8]. Taking into consideration that weight maintenance has been documented as a more challenging and vital goal than weight loss among overweight and obese individuals [9], several psychophysiological health factors may play a critical role in this long-term goal [10]. Noticeably, 20% of those who attempt to control weight are successful at long-term weight loss losing at least 10% of baseline body mass and maintaining the reduction for at least one year. Furthermore, high compliance with exercise programs appears to be the most successful approach for weight loss maintenance in overweight and obese individuals, underlining the vital role of regular exercise in the fight against the greatest global public health challenge of the 21st century [11]. 

### 1.2. Exercise: A Powerful Tool against Obesity

Regular physical activity and exercise have been extensively documented as a critical piece of the obesity management and treatment puzzle since physically active individuals impacted by being overweight or obese show higher physical and mental health levels compared with those who have the same body mass index (BMI) and are sedentary [12,13,14,15]. However, less than one in three adults demonstrates sufficient physical activity levels globally [16], and, therefore, sedentarism has been reported as a current major public health challenge since it is linked to the most prevalent cardiometabolic health-related diseases [17]. Hence, various exercise modes for people who are overweight or obese are currently underlined as some of the top trends in the health and fitness industry [18]. Specifically, the most updated exercise recommendations for these populations highlight the key role of combined (aerobic and resistance) training in weight loss and weight maintenance [14]. Additionally, recent evidence presents that combined training may be the most beneficial type of exercise for improving several cardiometabolic health-related markers in overweight and obese adults [15]. Nevertheless, combined training appears to be a time-consuming exercise approach [19], demonstrating high attrition and low compliance rates among adults with unhealthy weight [20]. On the other side, high-intensity interval training seems to be a time-efficient [21,22] and attractive [23] exercise modality. However, such a physically and mentally demanding strategy is not widely feasible and popular to overweight and obese individuals due to the prescribed exercise intensity levels [24]. However, time-efficient training programs may demonstrate high attractiveness, given that lack of time has been broadly documented as the number one exercise barrier among adults [25,26,27]. In addition, mind–body practices such as Pilates, yoga, tai chi, and qi gong are growing trends in the fitness industry and especially in the United States [28,29]. However, it is unclear whether all these practices including meditative and physical elements are efficient for improving various health, performance, and well-being markers among overweight and obese individuals.

### 1.3. Yoga Interventions and Obesity

Yoga is a mind–body exercise form defined as a group of mental, physical, and spiritual disciplines, which originated in ancient India and has changed over time. In Western societies, this alternative exercise mode focuses on physical postures, breathing techniques, and sometimes meditation or relaxation derived from yoga to enhance physical and emotional well-being [30]. Yoga practice is characterized mainly by floor-based poses widely known as asanas. There are many types of yoga such as Hatha, Vinyasa, Ashtanga, Iyengar, Kundalini, Kripalu, Yin, Bikram, Restorative, and Prenatal. Each type of yoga meets different expectations and requires different levels of mobility and functionality [31]. Typically, yoga in its ancient form placed a lot of attention on mental focus and spiritual energy and not on physical fitness. On the other hand, modern yoga emphasizes poses designed to stimulate inner peace and physical energy [30]. One of the fundamental training characteristics of yoga is flow, aiming to link each physical posture with the appropriate breath in order to ensure flow in every pose and transition. Such a flow strategically activates the full body without exhausting the involved muscles, providing a feasible and safe exercise approach to trainees without experience with this training method. [31]. As with any other exercise mode, yoga should be implemented carefully under the instruction of a qualified teacher. Deconditioned, sedentary, and beginners who are overweight or obese should avoid extreme and forceful poses (e.g., headstand, lotus position, and forceful breathing) in order to engage in a positive exercise experience [32,33,34,35].

**Figure 1:**
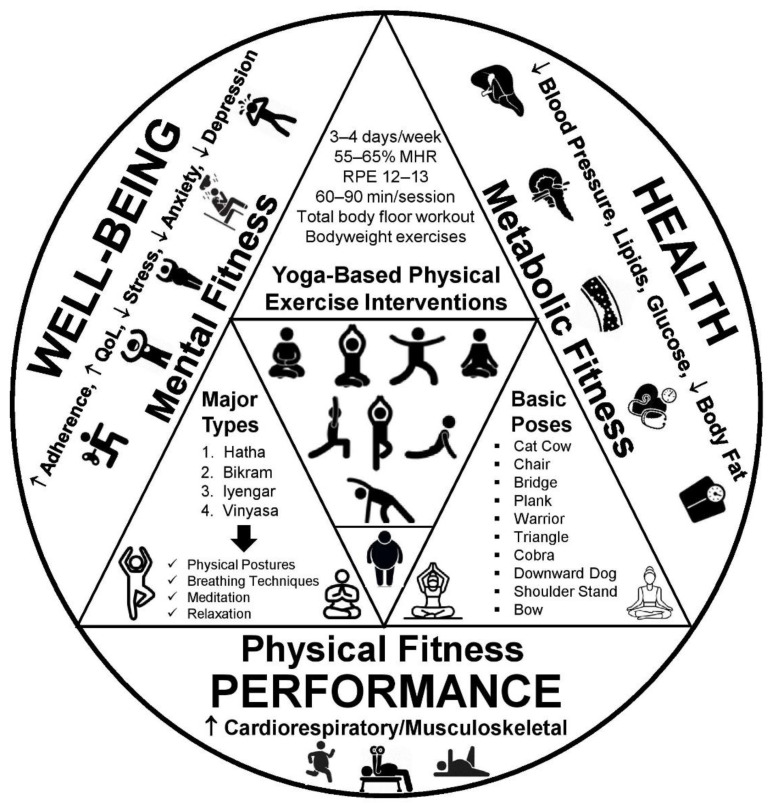
The psychophysiological effects of yoga interventions in overweight and obese individuals. MHR: maximum heart rate; RPE: rating of perceived exertion. [34,43,46,56,57].

Notably, yoga is considered one of the top trends among key fitness industry stakeholders around the globe, according to the latest regional [36,37,38,39] and global surveys [40]. Moreover, there is accumulating evidence showing that yoga may be beneficial for improving body composition [41,42,43,44], physical fitness [45,46], cardiovascular risk factors [47,48,49,50,51,52,53,54], chronic pain [55,56], and mental health [57,58,59,60,61,62,63] in various populations, including the obese. Therefore, yoga has been defined as a component of integrated therapeutic or preventive interventions supporting several populations suffering or being at risk of developing the most common lifestyle-related chronic conditions [64,65,66]. It is worth noticing that yoga seems to be a feasible [67], safe [34,35], and effective exercise approach for improving some weight-related outcomes [42,44] and energy balance parameters [43] in overweight and obese individuals. Thus, yoga is frequently prescribed to untrained people affected mainly by musculoskeletal and mental health issues due to its focus on low- to moderate-intensity full body movements, promoting trunk muscles activation, mobility, and functional capacity as well as reducing muscle stiffness [45,46], chronic pain [56], stress [62], and anxiety [63]. Previously inactive individuals with unhealthy weight belong to those populations and, thus, yoga appears to be a useful piece of the exercise programming puzzle for the overweight and obese seeking health and fitness benefits [31]. Postural deviations, movement dysfunctions, and low cardiorespiratory fitness are common among people who are overweight or obese [5,7]. Interestingly, yoga interventions present low injury rate as well as mild and adverse events in various age groups [32,33,34,35]. Taking this into consideration, yoga may be a user-friendly and pain-free exercise strategy for this underserved population that is likely to exercise in private settings, seeking facilities offering client-centered services and customized fitness training programs [28,68]. This is an important observation given that the vast majority of those who regularly practice yoga in the United States stated that health improvement and weight management are clearly the primary reasons for engaging in yoga interventions [69,70,71,72]. Figure 1 summarizes the effects of yoga interventions on cardiometabolic health, physical performance, and well-being in overweight and obese individuals [34,43,46,56,57]. 

The purpose of this brief review was to summarize the research methods applied and the main findings presented in yoga practice studies where selected physiological (e.g., anthropometric, body composition, physical fitness, and cardiometabolic health-related markers) and psychological outcomes (e.g., adherence, exercise enjoyment, depression, anxiety, and quality of life) were investigated. Such a review article may circulate the main outcomes on psychophysiological adaptations to yoga interventions in overweight and obese adults, aiming to identify research issues, considerations, and gaps in the literature. Since there is a lack of robust evidence on physiological and psychological responses to yoga interventions among populations with unhealthy weight, all relevant studies were retrieved, regardless of their methodology, in order to distribute a wide-ranging point of view on the current evidence. The present topical review addressed the general question: ‘What is known from published research about selected psychophysiological responses to numerous yoga-based physical exercise interventions in overweight and obese individuals?’

## 2. Materials and Methods

### 2.1. Literature Search Strategy

The PubMed/MEDLINE database was used to retrieve relevant articles from inception up to 1 October 2022 after a systematic electronic search by the author (A.B.) and a research assistant (A.K.). Additional searches were performed on Google and Google Scholar, because some articles on yoga are not listed in PubMed/MEDLINE. The search algorithm used the following terms: overweight, obese, obesity, and yoga. The complete search strategy is provided in the online Supplementary Materials (Table S1). Reference lists from articles were searched to retrieve additional potentially eligible articles.

### 2.2. Eligibility Criteria

Studies were considered eligible for inclusion if the following criteria were met: (1) participants were individuals of any age with no diagnosed comorbidities, signs/symptoms of any noncommunicable disease or eating disorders, or pregnancy, and with a BMI ≥25 kg/m^2^; (2) included studies employed an intervention of various types of yoga; and (3) investigated at least one of the following physiological and psychological outcomes: body weight, BMI, body fat percentage, lean body mass, waist circumference, waist-to-hip ratio, blood glucose, blood lipids, blood pressure, inflammatory and oxidative stress markers, cardiorespiratory fitness, muscular strength, muscular endurance, flexibility, balance, mobility, functional capacity, adherence, exercise enjoyment, affect valence, mood state, depression, anxiety, stress, or quality of life. All studies were required to be written in English and published in a refereed journal from inception up to 1 October 2022. 

As for the exclusive criteria, studies were not considered eligible for inclusion if the following criteria were met: (1) studies recruited a mixed sample of overweight/obese individuals with other chronic diseases per intervention arm; (2) articles where the effects of yoga intervention cannot be isolated because it was involved as part of a multicomponent lifestyle intervention or treatment in conjunction with medication, diet, or supplementation; (3) articles that did not assess the selected outcome measures; (4) studies published in a non-English language; and (5) studies that had not undergone full peer review (e.g., conference proceedings, posters, published abstracts, lay articles, proposed studies, dissertations, theses, reviews, commentaries, and debates).

### 2.3. Study Selection 

**Figure 2:**
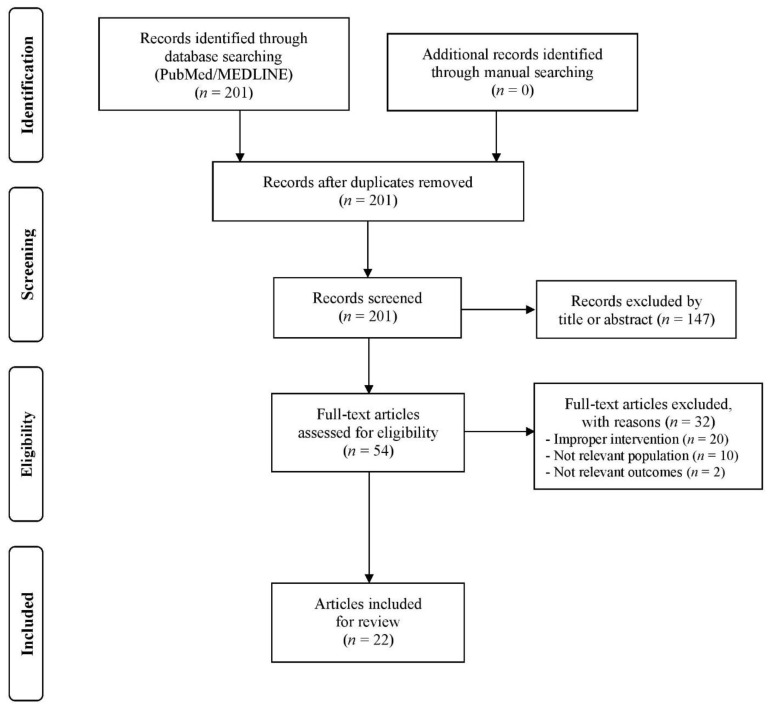
Flowchart of the systematic literature search.

The author (A.B.) and a research assistant (A.K.) independently screened the titles and abstracts of potentially eligible studies and downloaded the full texts of the remaining articles to assess their eligibility. Any discrepancies between the two assessors were resolved by discussion and consultation with a research fellow (V.K.). An intra-rater reliability of 90.9% was reported. EndNote X9 (Clarivate Analytics, Philadelphia, PA, USA) literature management software was used to manage the literature search records. The flow diagram is illustrated in Figure 2, showing the literature search and selection process in detail.

### 2.4. Data Extraction

The author (A.B.) and a research assistant (A.K.) independently extracted data using Microsoft Excel. Any disagreements were resolved by consensus. In the case of insufficient information, the authors of the included studies were contacted via email for missing values, where required. Data extraction included first author, year of publication, country, intervention duration, sample size, participant demographics (e.g., gender, mean age, and activity level), study design, yoga type, yoga intervention details (frequency, intensity, time, and type), and critical psychophysiological outcome measures and findings reported from each eligible study, as shown in Table 1. 

## 3. Results

### 3.1. Articles Retrieved

The electronic search returned 201 articles. After screening titles, abstracts, and full texts, 22 eligible studies were included in this review (Figure 2). Relevant data extracted from each article are presented in Table 1. 

### 3.2. Article Characteristics

Articles were published from 2012 to 2022 (2012–2016: *n* = 12 (55%) and 2017–2022: *n* = 10 (45%)), and research was conducted in seven countries (Asia: *n* = 13 (59%), North America: *n* = 7, (32%), Europe: *n* = 1 (4.5%), and Oceania: *n* = 1 (4.5%). There was a total sample of 1178 participants (56% female) who were overweight or obese across all studies that emphasized various ages (children/adolescents: *n* = 3 (13.5%), young adults: *n* = 12 (55%), middle-aged adults: *n* = 6 (27%), and older adults: *n* = 1 (4.5%). One (4.5%) article reported on the investigation of acute responses as a primary outcome and 21 (95.5%) articles reported on investigations of chronic responses (≥2 weeks) to yoga interventions. Training studies lasted from 1 to 48 weeks in duration (≤12 weeks: *n* = 14 (68%) and >12 weeks: *n* = 7 (32%)), with exercise session frequency ranging from two to six times per week and using quantitative methods. Articles reported on studies that implemented randomized controlled trials (*n* = 15, 68%), within-subject (*n* = 5, 23%), between-subject (*n* = 1, 4.5%), and case-control (*n* = 1, 4.5%). Articles reported on studies that assigned supervised (*n* = 19, 86.5%) or semi-supervised (*n* = 3, 13.5%) yoga interventions. Articles reported on studies that were conducted in a lab-based (*n* = 1, 4.5%) or field-based (*n* = 21, 95.5%) environment.

### 3.3. Exercise Protocols

Of the 22 yoga training protocols implemented in the reviewed studies, the following five different types of yoga were used: Hatha (*n* = 13, 59%), Bikram (*n* = 5, 23%), Iyengar (*n* = 2, 9%), and Vinyasa (*n* = 2, 9%). The authors presented details of the yoga interventions regarding the prescribed training parameters (e.g., frequency, time, and type) but not the exercise intensity since the large majority of eligible studies (*n* = 19, 86%) did not report data related to the mean percentage of the maximum heart rate and the mean rating of perceived exertion of participants. The most frequently reported yoga training protocol was a supervised Hatha-based intervention (3–5 sessions/week, 60–90 min/session) conducted in a real-world setting.

## 4. Discussion

### 4.1. Summary of Main Results

Through a topical review, 22 articles were systematically retrieved that published psychophysiological outcomes associated with yoga practice in sedentary individuals who were overweight or obese. The majority (59%) of eligible studies included in this review investigated Hatha-based yoga protocols in a supervised (87%) and field-based setting (96%). The finding that 45% (10/22) of the eligible articles were published between 2017 and 2022, while 5 studies (23%) were published in 2022, highlights the novelty of this particular emerging research topic. This mini-review summarizes the current literature to support further discussion of the questions, reflections, and gaps that need to be considered in future studies. Overall, yoga-based physical exercise interventions seem to be a beneficial exercise strategy for individuals who are overweight or obese, mostly seeking to improve anthropometric, body composition, physical fitness, and cardiometabolic health variables. As for mental-health-related indicators, no robust data were found across any eligible studies examining these particular responses to yoga practice in overweight and obese persons. Moreover, the significant lack of evidence in all eligible studies concerning adverse events does not support the investigation of safety and feasibility of yoga-based physical activities in real-world conditions for overweight and obese people. Additionally, research with an emphasis on the impact of yoga on several psychophysiological markers in overweight and obese older adults is scarce.

### 4.2. Body Composition

Anthropometric and body composition variables have been the most frequently investigated physiological outcomes in the yoga-based training literature for individuals who are overweight or obese. On the other side, there is a serious lack of research to identify whether psychological outcomes can be achieved by people with unhealthy weight following various yoga-based physical exercise interventions. However, yoga has been broadly recognized as one of the most popular fitness services globally [28,40]. The core results of this mini-review demonstrate that yoga users achieved noticeable reductions in body weight, BMI, body fat, waist size, waist-to-hip ratio, lean body mass, bone mineral density, and bone mineral content. This is an important outcome showing the beneficial role of yoga practice in weight and fat loss. The most frequently implemented weekly exercise volume of 180–360 min (3–4 sessions/week, 60–90 min/session) across all eligible studies included in this topical review appears to be an efficient approach for eliciting positive alterations in energy balance through increased energy expenditure. Such a physiological adaptation may be a valuable tool for inactive, non-dieting persons who are overweight or obese [14,95]. Considering that the most commonly recommended exercise strategy to those with unhealthy weight requires at least 150–300 min of moderate to vigorous intensity per week, it appears that yoga as an alternative exercise mode meets the suggested weekly exercise volume for this population [14,96]. 

Since yoga-based practice mainly incorporates bodyweight movements into a full-body, resistance-based floor workout [97], it seems possible to improve muscular fitness [98], crucially contributing to positive changes in several anthropometric and body composition parameters among sedentary overweight and obese individuals. It has been reported that yoga at varying tempos can meet or exceed moderate-intensity exercise recommendations (3.0–5.9 metabolic equivalents (METs)) [14], serving as an alternative, low-impact training solution for overweight and obese populations with no prior exercise experience and poor cardiorespiratory and musculoskeletal fitness levels [99]. However, a 56-min Hatha yoga session provoked lower responses in exercise intensity (49.4% MHR, RPE 12–13) and energy expenditure (3.2 kcal/min, 2.5 METs) compared with the above suggested levels in healthy young adults with at least one year of experience in yoga practice [100]. Additionally, a 35-min Hatha yoga session performed by healthy young adults who had been practicing yoga for 6–10 years, showed similar exercise intensity (52.3–54.5% MHR) and energy expenditure (1.3–2.5 kcal/min, 1.0–2.2 METs) [101]. On the other side, a 60-min Vinyasa yoga session resulted in higher mean exercise intensity (58% MHR, RPE 13–14) and energy expenditure (4.6 kcal/min, 3.6 METs) compared with the two aforementioned Hatha-based yoga interventions [102]. 

In general, yoga demonstrates metabolic demands that are comparable to those observed in adults when performing recreational aerobic training, such as brisk walking at a self-selected pace [99,102]. Given also that typical yoga practice involves three main types of activity, namely static physical postural, breathing techniques, and meditation [103], it is obvious that each yoga style may be characterized by different metabolic demands depending on its focus on the above-mentioned activities. In summary, yoga practice may induce a favorable effect on certain anthropometric and body composition parameters in overweight and obese individuals due to metabolic efficiency and energy expenditure during exercise sessions on a regular basis. However, the inclusion of yoga practice in a weight management program may help reduce energy intake and improve eating habits in several ways that cannot be explained here [43], but there is not sufficient evidence to recommend that yoga interventions alone can lower energy expenditure. Additionally, there are currently inconsistent results that yoga is associated with elevated non-yoga physical activity levels [43]. Hence, the overall body of literature presents insufficient evidence to indicate the effectiveness of yoga on energy intake and physical activity among obese individuals. Such an outcome may be supported by the fact that this mind–body exercise option seems to be beneficial for improving physical function correlated with body composition improvements [45,46]. However, the promising preliminary evidence that yoga can lead to improvements in anthropometric and body composition variables should be further investigated through more large-scale, rigorously designed randomized controlled trials.

### 4.3. Cardiometabolic Health

Cardiometabolic health complications are frequently present among people who are overweight or obese [4,5] and, thus, a comprehensive examination of the efficacy of yoga practice on numerous physiological health markers may be critical for detecting the role of this mind–body exercise mode in the overweight and obese. It has been well reported that people with unhealthy weight are predisposed to the development of lifestyle-related chronic diseases, primarily due to sedentarism, high levels of visceral fat, chronic inflammation, and impaired redox status [4]. Recent evidence demonstrates that several exercise types elicit favorable changes in various cardiometabolic health indicators in persons who are overweight or obese [15,104]. In terms of yoga, research emphasizing overweight and obese individuals in this particular area of outcome measures is scarce. However, both short- and long-term yoga interventions appear to lower cardiovascular risk factors in various populations [105,106,107,108], while the present brief review shows inconsistent results regarding the effects of yoga practice on vital cardiometabolic health-related indices in populations who are overweight or obese [77,79,90,92]. 

In general, the effectiveness of such an alternative mind–body exercise mode on important health markers linked to various lifestyle-related chronic diseases among sedentary overweight and obese adults has been poorly examined, and there are no strong proofs concerning the comparative effectiveness of different exercise modes in this research area [97,98]. However, the main findings exhibited here are partially in line with those reported for individuals with poor glycemic control and lipid profile [49,51,52,53,109,110], raised blood pressure [48,50], and cardiovascular disease [54] following various yoga-based interventions. It is difficult to explain such results since mechanistic studies investigating the role of yoga practice in health are very limited. However, the improvements in resting cardiovascular function markers such as heart rate and blood pressure promotes an altered autonomic balance in yoga users due to the predominant parasympathetic system and relatively reduced sympathetic tone [107]. This may be explained by the beneficial effects of yoga on the autonomic nervous system activity through a reduction in basal sympathetic tone and a rise in basal parasympathetic activity. Such a modulation of autonomic nervous system can also be associated with significant improvements in various key mental health markers such as stress, anxiety, and depression, eliciting positive alterations in arterial tone and peripheral resistance, resulting in improved blood pressure and resting heart rate [107]. A possible reason behind the present beneficial alterations in blood glucose and lipid metabolism markers is that yoga practice reduces muscle insulin resistance and increases glucose disposal through various mechanisms with or without weight loss [109]. However, these findings should be taken into consideration cautiously, since the number of selected trials in this brief review is limited. Thus, further research is warranted to investigate whether yoga-practice-induced adaptations are beneficial for metabolic health in obese individuals. Taking this into account, yoga could be a potential component of a comprehensive therapy of lifestyle and related risk factors for chronic diseases. Such a complementary therapeutical role should be investigated further, aiming to identify the beneficial long-term effects of yoga on the most common impaired cardiovascular disease risk factors affected by the inactivity and obesity epidemics [111]. 

### 4.4. Physical Fitness

Modern yoga is most often linked to physical practice of various postures often weaved together in different styles [103], and, therefore, yoga practice is a multimodal activity and not a cardiovascular exercise mode. However, it may increase maximal aerobic capacity in middle-aged adults with two years of experience in yoga compared with active individuals who participated in regular aerobic exercise according to the current guidelines for cardiovascular disease prevention [112]. Such a finding may be supported by the strengthening of respiratory muscles due to the dynamic inspiration and expiration during yoga practice [113]. Overall, the lack of robust evidence supporting the positive role of yoga in cardiorespiratory fitness in several populations, including the obese, is one of the main observations produced by the present review. Recent data show that this mind–body exercise type is not able to elicit beneficial changes in cardiorespiratory fitness in older adults [46]. Since yoga mostly combines static and dynamic poses in conjunction with breathing techniques, it promotes not only total body activation through bodyweight movements with a great range of motion but also relaxation without reaching muscle exhaustion [30,31]. Thus, such an exercise option enhances significant increases in muscular endurance, flexibility, balance, coordination, mobility, and posture [45,46] while promoting an injury-free activity for various populations [32,33,34,35]. Taking this into consideration, it appears that this alternative exercise strategy may reduce the risk for chronic pain [56] that is frequently present among insufficiently active individuals who are overweight or obese due to their poor body functionality and numerous physical limitations [7]. 

Yoga practice seems to be efficient for ameliorating important motor fitness parameters supporting spine health and increasing physical performance in daily tasks [45,46]. This finding is important, given that overweight and obese individuals are unlikely to demonstrate an ideal posture with no movement dysfunction [114,115,116]. Of particular importance is that yoga-based physical exercise interventions show beneficial alterations in physical–functional performance in overweight/obese older women following an 8-week Hatha-based protocol [94]. Additionally, data from an 8-week Hatha yoga intervention indicates that such a physical practice is just as effective as stretching–strengthening exercises in elevating functional fitness levels in sedentary, healthy older adults [117]. For these reasons, such an alternative exercise approach incorporating static and dynamic muscle-strengthening movements as well as breathing exercises into a full body workout may provide overweight and obese people with an inclusive environment and substantial improvements in activities of daily living [118]. Interestingly, yoga has been reported as an effective tool for reducing chronic low back pain, since it enhances core strength, motor and posture control, and full body stability, playing a vital role in lumbar spine health [55]. Such results may be explained by the fact that a typical yoga session consists mostly of movement-based activities incorporating pain-free postures with a great range of motion into an exercise space, but with no local muscle fatigue. This is a user-friendly exercise strategy promoting bodily integrity and motor task performance among inactive individuals with high BMI.

Overall, yoga practice may be a useful component of a comprehensive treatment approach for those affected by the most prevalent musculoskeletal health issue among adults in the Western world [118]. However, in this mini-review, only one study investigated physical fitness parameters such as muscular strength, flexibility, functional mobility, balance, and aerobic capacity [94], underlining the scarcity of research regarding the effectiveness of yoga practice on physical and motor fitness variables in overweight and obese individuals. Such an observation may support the opinion that yoga is not just another type of exercise. Instead, yoga is mostly a multifaceted spiritual tool able to induce enhanced health and well-being to its regular users [103], and, thus, typical exercise modes and physical components of yoga practices demonstrate several similarities but also important differences [119]. Taking these facts into account, it does not appear clear whether yoga can elicit meaningful improvement in a broad spectrum of musculoskeletal fitness variables following short- or long-term interventions in these populations compared with other popular neuromuscular exercise modes [120,121,122]. Thus, further research is needed to identify whether this mind–body activity can be widely adapted for individuals with lower levels of functioning or disabilities.

### 4.5. Mental Health

Given that mental-health-related disorders are common among individuals who are overweight or obese [12,123,124,125,126], an in-depth study of the efficacy of various physical exercise types, including yoga-based interventions on numerous psychological health indices, may be important for assessing the vital role of exercise training in the prevention, management, and treatment of the overweight and obese [12,123]. Recent data indicate that physical exercise can promote beneficial changes in quality of life, which is an important mental health indicator [12,123]. In this review, 41% of eligible studies included at least one measure outcome related to mental health. Additionally, the impact of yoga practice on mental health among various populations appears as an emerging area of interest [57,58,59,60,61,62,63], considering that a typical yoga intervention engages not only a physical component (dynamic poses) but also specific breathing techniques and meditation in order to enhance relaxation, calmness, and stress relief [30,31]. Significant changes were found in key psychological health markers across all relevant studies included in this brief review. Such results show that yoga-based physical intervention may be an impactful tool for improving anxiety, depression, mood, stress, and quality of life in previously inactive people who are overweight or obese. 

Interestingly, the role of physical activity interventions has been poorly studied in this cohort. Thus, there is no strong evidence regarding the comparative effectiveness of different exercise types on this particular research area [97]. Noticeably, experienced yoga users with obesity demonstrate higher quality of life levels than those with no experience, showing that this mind–body exercise strategy may enhance specific mental health aspects in this cohort [127]. According to the American Osteopathic Association, the incorporation of meditation and breathing into a yoga session may enhance regular users’ mental well-being by creating mental clarity and calmness, increasing body awareness and reducing chronic pain and stress, and it relaxes the mind and improves concentration. Hence, yoga appears to be a cost-effective component of an adjunct therapy implemented as a self-care behavioral strategy, aiming to elevate several aspects of mental health in various populations, including those who are overweight or obese [128]. In this topical review, the vast majority of eligible studies (8/9, 89%) investigating various psychometrics focused on anxiety, depression, stress, and quality of life. Noteworthy, all of those studies demonstrated positive alterations in selected psychological-health-related markers among overweight and obese adults [73,74,75,78,80,81,87,88,93]. These findings are in agreement with those reported for other populations, supporting the beneficial role of yoga practice in lowering anxiety, depression, stress [129], and professional burnout [130]. In general, the improvements in well-being through lower anxiety and depression levels have been considered as an important research area. Therefore, key components of yoga interventions for reducing these particular mental health markers have been established due to lack of detail and consistency of approach of yoga-based physical exercise interventions [131]. However, only one eligible article studied the effects of yoga practice on exercise enjoyment [93] while no eligible studies were found investigating affective valence and adherence. Given the significant lack of data regarding the yoga-practice-induced improvements in specific aspects of mental health, it does not seem clear whether such a type of mind–body exercise is efficient to induce substantial alterations in psychological-health-related markers of overweight and obese individuals. 

Of particular importance is that distress tolerance was positively associated with adherence among overweight women following two weekly Bikram yoga classes for 8 weeks in a real-world gym setting [132]. Interestingly, 86% of eligible studies reported low attrition rates ranging from 0% to 16% and implemented a supervised yoga intervention in a field-based setting. Such an observation may indicate the importance of supervision by trained yoga teachers who ensure an inclusive and safe environment that may promote adherence to regular yoga practice among overweight and obese individuals. Moreover, weekly time commitment may be also an important factor affecting compliance with yoga practice. In this review, the most frequently reported yoga training protocol required 180–450 min per week (3–5 sessions, 60–90 min/session). Given that lack of free time has been considered the number one barrier to physical activity among adults [25,26,27], such a high weekly training volume may not be able to provide regular yoga users with an engaging and feasible exercise experience. Furthermore, overweight and obese adults show high attrition and low adherence rates when participating in physical activity and various types of structured exercise [20]. However, time-efficient training modalities appear to be attractive to this population despite the higher metabolic demands required [133,134] compared with other popular exercise types, such as aerobic and resistance training [20] as well as Pilates [122]. According to the findings presented here, 32% of eligible studies implemented long-term (>12 weeks) interventions, and, therefore, low compliance rates observed in these studies cannot be considered robust evidence for the impact of yoga-based physical exercise interventions on adherence to this alternative practice. Importantly, regular exercise incorporating not only aerobic but also muscle-strengthening components into a single session or separately in different sessions are suggested at least twice per week for overweight or obese people to promote health and fitness benefits [14,135]. Thus, yoga practice may be implemented as an adjunct bodyweight training for populations who are overweight or obese. 

In summary, controlled rhythmic breathing techniques widely used in yoga practice produce a reduction in basal sympathetic tone and an increase in basal parasympathetic activity. Additionally, the meditation and relaxation components of a typical yoga session modify the state of anxiety and lower stress-induced sympathetic overactivity, resulting in reducing arterial tone and peripheral resistance, which are responsible for decreasing diastolic blood pressure and heart rate at rest. Such functions promote better peripheral circulation and blood flow to tissues and downregulate the stress hormones levels resulting in meaningful improvements in various mental health indices [107].

### 4.6. Future Research

The small number of available randomized controlled trials provides weak evidence regarding the effectiveness of yoga interventions on selected physiological but mostly psychological outcome measures in overweight and obese individuals. Overall, the feasibility of yoga as a mind–body exercise mode for this cohort is not very well established. Further research is warranted to identify whether this alternative exercise approach can play a positive role in a comprehensive training plan for sedentary overweight and obese individuals. However, from a physiological perspective, the current data support the viability of yoga as an alternative bodyweight exercise strategy, given that it shows beneficial changes in numerous indicators among people who are overweight or obese. More research is needed on this area, focusing on the dose–response effects of long-term yoga interventions (>12 weeks) on various physical performance and cardiometabolic-health-related markers for overweight and obese individuals in real-world conditions. Furthermore, the potential mechanisms behind positive alterations in numerous psychophysiological health indices should be investigated in the future through larger samples and high-quality randomized controlled trials conducted in a real-world setting.

Since evidence regarding the effectiveness of yoga-based physical interventions on vital mental-health-related markers such as anxiety, depression, mood state, quality of life, exercise enjoyment, affect valence, and adherence are currently limited for overweight and obese people, the findings briefly summarized here may help researchers investigate yoga interventions promoting beneficial alterations in selected psychophysiological indicators in previously inactive individuals with unhealth weight. Such a mind–body exercise type may be a valuable strategy to explore how the large majority of the adult population worldwide regularly engage in adapted yoga interventions characterized by supervision, progression, and variety. In addition, more randomized controlled trials with a focus on dropout rate and adherence to yoga practice are needed, given that yoga has been documented as one of the current top trends in the health and fitness industry, especially in women [28,40,136,137,138]. Furthermore, data related to the impact of yoga on several musculoskeletal (e.g., muscular strength, muscular endurance, flexibility, mobility, balance, and functional capacity) and cardiorespiratory fitness parameters are lacking across all eligible studies presented here. In this review, adverse events to yoga practice were not clearly reported in the majority of eligible studies. Given that regular yoga practice has been documented as a feasible and safe physical exercise strategy for the general population [32,33,34,35]; however, pain, soreness, muscle injuries, and fatigue have been reported as the most often adverse effects among yoga-experienced people [139,140]. This is an important observation highlighting the need of additional studies examining whether yoga provides an inclusive environment in order to identify whether this alternative exercise mode is effective, feasible, and popular to the masses affected by being overweightness or obese.

This brief review shows that yoga training program configuration should be a priority in future research attempts, aiming to support exercise professionals with insights into various training parameters as previously reported [141]. It is worth noticing that details concerning the exercise protocol development (e.g., intensity, volume, progression, and overload) were scarce across all eligible studies included in this review. From a methodological perspective, short-term yoga-based lifestyle interventions incorporating yoga practice into a multicomponent treatment conducted in a small group manner have been frequently studied among overweight and obese adults. Such an integrated approach demonstrates significant improvements in various cardiometabolic and mental health indicators [67,142,143,144,145,146]. However, this particular methodology cannot provide robust evidence proving the efficacy of stand-alone yoga practice, since prescribed diet and nutritional advice are commonly fundamental components of yoga-based lifestyle interventions that significantly affect various health-related outcomes and weight management in this cohort [147]. In addition, a recent scoping review revealed that systematic reviews of exercise and physical activity interventions for health-related outcomes do not consistently make clear whether they include or exclude yoga as a form of exercise [66]. This is an important finding that should be carefully considered by authors conducting future attempts to this particular research area, focusing on targeted systematic reviews and meta-analyses. 

### 4.7. Strengths and Limitations

This brief review has several strengths: (i) the rigorous study selection process; (ii) the classification of yoga types administered across all studies; (iii) the provision of practical recommendations for future research in this particular area; and (iv) the summary of important outcomes regarding the psychophysiological adaptations to yoga interventions in overweight and obese populations who represent almost two in three adults in the Western world. However, the present study has numerous limitations: (i) the lack of assessment of the quality of data presented in all eligible studies included in the review; (ii) the synthesis of available data was not addressed since existing evidence was classified narratively; (iii) the great methodological diversity observed among yoga interventions concerning the program design and training parameters that were implemented across all eligible studies included in this review; (iv) the emphasis of the present topical review was mainly on a variety of study designs and methodologies, and, thus, it was not focused on a comprehensive analysis of a smaller number of randomized control trials; and (v) the selection of one database for searching relevant articles rather than multiple databases and grey literature. 

## 5. Conclusions

Yoga is a popular mind–body practice using floor-based bodyweight movements, breathing techniques, meditation, and relaxation, promoting psychophysiological adaptations associated with improved anthropometric parameters, body composition, physical fitness, cardiometabolic health, and mental health indicators in overweight and obese individuals. Yoga-based physical exercise interventions have not been documented as intensively as other conventional training modalities, such as aerobic and resistance training or even Pilates, for overweight and obese individuals. Taking this into account, yoga routines for those populations who demonstrate poor functional capacity and several physical limitations may be a feasible and injury-free exercise strategy for health, fitness, and well-being. In summary, the present review provides evidence suggesting that yoga can be an effective, safe, and enjoyable type of exercise for apparently healthy individuals struggling with being overweight or obese. However, long-term yoga interventions conducted in free-living conditions through high-quality randomized controlled trials are warranted in order to investigate the real-world impact of yoga on various psychophysiological health indices in both overweight and obese men and women of all ages. Given that yoga, as an alternative form of exercise, can be included within a standard behavioral weight management intervention, current exercise prescription guidelines should consider including this practice in future recommendations for overweight and obese individuals. Such an approach may support a large majority of the world adult population impacted by the inactivity and obesity epidemics that seem to be the greatest threats to public health and economy [148].

## Figures and Tables

**Table 1:** Data extracted from each article included for review.

Article (First Author, Year)	Country	Duration (Weeks)	Sample ^1^ N/F/M	Mean Age ± SD (Years)	Activity, BMI Classification	Study Design	Yoga Type	Yoga Intervention Characteristics ^2^	Findings	Dropout Rate ^3^
Braun (2012) [73]	United States	1	31/31/0	32.0–65.0	Inactive, Overweight/ Obese	Acute, Within- Subject	Hatha	5 sessions/week, 90 min/session, supervised, field-based	SRE (↓); Mood (↑)	16%
Cramer (2016) [74]	Germany	12	60/60/0	47.8 ± 9.8	Inactive, Overweight/ Obese	Chronic ^4^, RCT	Hatha	2 sessions/week, 90 min/session, supervised, field-based	BW, BMI, BF, WHR, SRE (↓); LBM, QoL (↑)	7%
Dhananjai (2013) [75]	India	24	272/124/148	33.9 ± 7.7	Inactive, Obese	Chronic, Case- Control	Hatha	5 sessions/week, 60 min/session, supervised, lab-based	BW, BMI, WC, WHR, ANX, DEP (↓)	0%
Forseth (2022) [76]	United States	12	18/9/9	11.5 ± 1.7	Inactive, Obese	Chronic, Between-Subject	Hatha	1 session/week, 45–60 min/session, supervised, field-based	BMI (↓)	40%
Guo (2014) [77]	China	48	50/50/0	36.8 ± NR (18.0–48.0)	Active, Overweight	Chronic, Within- Subject	Bikram	4 sessions/week, 90 min/session, supervised, field-based	BW, BF, WC, TC, LDL, TG, RHR, SBP, DBP (↓); HDL (↑)	0%
Hainsworth (2018) [78]	United States	8	9/5/4	14.0 ± NR (11.0–17.0)	Inactive, Overweight/ Obese	Chronic, Within- Subject	Iyengar	2 sessions/week, 60 min/session, supervised, field-based	QoL (↑)	10%
Hewett (2017) [79]	Australia	16	56/44/12	37.2 ± 10.8	Sedentary, Overweight/ Obese (83%) and Normal Weight (17%)	Chronic, RCT	Bikram	3–5 sessions/week, supervised, field-based	BW, BMI, WC, LBM, BF, TC, LDL, HDL, TG, BG, RHR, DBP, SBP (↔)	11%
Hopkins (2016) [80]	United States	8	39/39/0	33.5 ± 6.4	Inactive, Overweight/ Obese	Chronic, RCT	Bikram	2 sessions/week, 90 min/session, supervised, field-based	SRE (↓)	25%
Hunter (2016) [81]	United States	8	43/35/8	37.6 ± 11.0	Sedentary, Overweight/ Obese	Chronic, Within- Subject	Bikram	3 sessions/week, 90 min/session, supervised, field-based	QoL (↓)	35%
Hunter (2013) [82]	United States	8	29/23/6	39.2 ± 11.0	Sedentary, Overweight/ Obese	Chronic, Within- Subject	Bikram	3 sessions/week, 90 min/session, supervised, field-based	BW, BMI, BG (↓)	0%
Jorrakate (2015) [83]	Thailand	4	16/8/8	22.2 ± 1.4	Sedentary, Overweight/ Obese	Chronic, RCT	Hatha	3 sessions/week, 45 min/session, supervised, field-based	Balance (↑)	0%
Lee (2012) [84]	South Korea	16	16/16/0	54.5 ± 2.8	Sedentary, Overweight/ Obese	Chronic, RCT	Hatha	3 sessions/week, 60 min/session, supervised, field-based	BW, BMI, WC, BF, VFA, TC, LDL, TG, SBP, DBP, BG, INSL, HOMA-IR (↓); ADPN, HDL (↑)	0%
Na Nongkha (2021) [85]	Thailand	12	40/40/0	20.0 ± 0.8	Inactive, Overweight/ Obese	Chronic, RCT	Vinyasa	3 sessions/week, 50 min/session, 65–75% MHR, supervised, field-based	BW, BMI, BF (↓); LBM (↑)	0%
Na Nongkha (2022) [86]	Thailand	12	60/60/0	18.0–22.0	Inactive, Overweight	Chronic, RCT	Vinyasa	3 sessions/week, 45 min/session, 65–75% MHR, supervised (online), field-based	BW, BMI, WC, RHR (↓)	0%
Rshikesan (2016a) ^5^ [87]	India	14	72/0/72	41.1 ± 10.4	Sedentary, Overweight/ Obese	Chronic, RCT	Hatha	5 sessions/week, 90 min/session, supervised, field-based	BW, BMI, BF, WC, SRE (↓)	10%
Rshikesan (2016b) ^6^ [88]	India	26	72/0/72	41.1 ± 10.4	Sedentary, Overweight/ Obese	Chronic, RCT	Hatha	5 sessions/week, 90 min/session, semi-supervised, field-based	BW, BMI, BF, WC, SRE (↓)	10%
Rshikesan (2017) ^5^ [89]	India	14	72/0/72	41.1 ± 10.4	Sedentary, Overweight/ Obese	Chronic, RCT	Hatha	5 sessions/week, 90 min/session, supervised, field-based	BW, BMI (↓); BMC, BMD (↑)	10%
Seo (2012) [90]	South Korea	8	20/0/20	14.7 ± 0.7	Inactive, Obese	Chronic, RCT	Hatha	3 sessions/week, 60 min/session, supervised, field-based	BW, BMI, BF, TC (↓); LBM, RMR, HDL (↑); LDL, TG, BG, INSL, HOMA-IR (↔)	0%
Sharma (2022a) ^5^ [91]	India	12	65/19/46	37.9 ± 6.4	Sedentary, Overweight/ Obese	Chronic, RCT	Hatha	6 sessions/week, 60 min/session, semi-supervised, field-based	BW, BMI, WHR (↓)	10%
Sharma (2022b) ^5^ [92]	India	12	65/19/46	37.9 ± 6.4	Sedentary, Overweight/ Obese	Chronic, RCT	Hatha	6 sessions/week, 60 min/session, semi-supervised, field-based	NF-kappa B, TNF-α, IL-6 (↔)	10%
Unick (2022) [93]	United States	12	51/51/0	34.3 ± 3.9	Sedentary, Overweight/ Obese	Chronic, RCT	Iyengar	2 sessions/week, 60 min/session, supervised, lab-based	BW, SRE (↓); ENJ (↑)	6%
Widjaja (2021) [94]	Thailand	8	22/22/0	62.0 ± 1.0	Sedentary, Overweight/ Obese	Chronic, RCT	Hatha	3 sessions/week, 60 min/session, 52–60% MHR, supervised, field-based	Strength, Flexibility, Functional Mobility, Balance, CRF (↑)	0%

ADPN, adiponectin; ANX, anxiety; BG, blood glucose; BF, body fat; BMC, bone mineral content; BMD, bone mineral density; BMI, body mass index; BW, body weight; CRF, cardiorespiratory fitness; DBP, diastolic blood pressure; DEP, depression; ENJ, enjoyment; HDL, high-density lipoprotein cholesterol; HOMA-IR, homeostatic model assessment for insulin resistance; IL-6, interleukin-6; INSL, insulin; LBM, lean body mass; LDL, low-density lipoprotein cholesterol; MHR, maximum heart rate; NF-Kappa B, nuclear factor-kappa B; NR, not reported; QoL, quality of life; RCT, randomized controlled trial; RHR, resting heart rate; RMR, resting metabolic rate; SBP, systolic blood pressure; SRE, stress; TC, total cholesterol; TG, triglycerides; TNF-α, tumor necrosis factor-α; VFA, visceral fat area; WC, waist circumference; WHR, waist-to-hip ratio. ^1^ Sample size refers to participants who completed (not being recruited) the study; ^2^ Session duration (including warm-up and cool-down); ^3^ Dropout rate refers to participants who did not complete the yoga intervention; ^4^ Chronic responses (≥2 weeks); ^5^ Same trials, but outcome measures reported in two separate papers; ^6^ Same trials, but results for each time point reported separately. ↑ indicates higher; ↓ indicates lower; ↔ indicates unchanged.

## Supplementary Materials

The following supporting information can be downloaded at: www.mdpi.com/article/10.3390/diseases10040107/s1, Table S1: Search algorithms and results.
