# Supplementary material for: Psychophysiological Adaptations to Yoga Practice in Overweight and Obese Individuals: A Topical Review
Source: Diseases. 2022 Nov 17;10(4):107. doi: 10.3390/diseases10040107 (PMC9680480; doi:10.3390/diseases10040107)
Supplement: Supplementary file 1 [file diseases-10-00107-s001.zip › diseases-2016251-supplementary.pdf]

Table S1. PubMed/MEDLINE search algorithms and results.

Search	Query	Items found
6	Search (4 AND 5)	201
5	Search yoga[Title/Abstract]	6,159
4	Search (1 OR 2 OR 3)	381,295
3	Search overweight[Title/Abstract]	83,550
2	Search obese[Title/Abstract]	144,285
1	Search obesity[Title/Abstract]	302,702
